# Attitudes of pediatricians toward Children’s consumption of ionic beverages

**DOI:** 10.1186/s12887-018-1154-3

**Published:** 2018-05-25

**Authors:** Akihisa Okumura, Shinobu Ida, Masaaki Mori, Toshiaki Shimizu, On behalf of the Committee on Pediatric Nutrition of The Japanese Pediatric Society, Shinobu Ida, Setsuko Ito, Mikako Inokuchi, Toshihiro Ohura, Akihisa Okumura, Mitsuyoshi Suzuki, Kimitaka Takitani, Kazushige Dobashi, Mitsuhiko Hara, Toru Kikuchi, Shigetaka Sugihara, Nobuo Yoshiike, Tomoo Okadan, Kazunari Kaneko, Chiharu Tsutsumi, Yuriko Ohta, Keiichi Hanaki, Kazue Kawakami, Yoshio Hanawa, Hiroaki Inomata, Tatsuya Oguni, Atsuyuki Yamataka, Yuko Bito, Keiichi Uchida, Hiroko Kodama, Masaaki Mori, Toshiaki Shimizu

**Affiliations:** 1 Department of Pediatrics, Aichi Medical University, Nagakute, Japan; 2 Department of Pediatrics, Juntendo University Faculty of Medicine, Tokyo, Japan; 3 Department of Pediatric Gastroenterology and Endocrinology, Osaka Medical Center and Research Institute for Maternal and Child Health, Osaka, Japan; 4 Department of Lifetime Clinical Immunology, Graduate School of Medical and Dental Sciences, Tokyo Medical and Dental University, Tokyo, Japan

**Keywords:** Vitamin B1 deficiency, Ionic beverages, Excessive consumption, Attitude

## Abstract

**Background:**

The aim of our study was to clarify the attitudes of pediatricians toward children’s consumption of ionic beverages.

**Methods:**

A questionnaire survey of pediatric practitioners’ attitudes toward the consumption of ionic beverages was administered to 537 doctors under 60 years of age who were members of the Japanese Pediatric Society.

**Results:**

We received 215 valid responses from 182 board-certified pediatric specialists and 31 non-specialists. Approximately 60% of respondents recommended ionic beverages either often or sometimes. About half of all respondents cautioned patients about excessive consumption. About 40% had experienced at least one instance of excessive consumption characterized by acute symptoms including vomiting, diarrhea, and pyrexia. Specialists were more likely to recommend ionic beverages for oral rehydration than did non-specialists. Non-specialists more often recommended ionic beverages to patients with pyrexia.

**Conclusions:**

Pediatricians’ attitudes toward children’s consumption of ionic beverages were generally appropriate. Pediatric specialists’ attitudes were more appropriate than were those of non-specialists.

## Background

We have reported vitamin B1 deficiency (VB1D) caused by excessive consumption of ionic beverages in Japan [1]. The patients with VB1D were infants and young children who continued to drink a large amount of ionic beverages every day for several months, some of whom were raised in a non-nurturing environment [2–8]. VB1D caused by excessive soft drink consumption is presumed to be unique to Japan, where ionic beverages are popular and are widely used on a daily basis. It cannot be ignored that doctors’ recommendations of ionic beverages have triggered excessive consumption by some children with VB1D [1, 2]. Oral fluid replacement is widely accepted as important in patients with dehydration caused by infectious diseases such as acute viral gastroenteritis [9, 10]. Several suitable ionic beverages are commercially available in Japan [11, 12]. Appropriate oral fluid replacement is essential to treat and prevent dehydration in children. On the other hand, the annual sales of ionic beverages are increasing in Japan. Some promotions target children, potentially creating a risk of excessive consumption. Parents and caregivers must learn how to prevent excessive consumption by children. However, the repetitive occurrence of VB1D due to excessive ionic beverage consumption among children suggests that parents and caregivers may lack adequate knowledge.

Few studies have evaluated the attitudes of pediatricians toward children’s consumption of ionic beverages. The aim of the present study was to explore the attitudes of pediatricians toward consumption of ionic beverages; appropriate attitudes should help prevent excessive consumption. Thus, we surveyed the attitudes of pediatric practitioners toward consumption of ionic beverages.

## Methods

We identified 537 doctors who met the following four criteria: 1) membership in the Japanese Pediatric Society; 2) working in Tokyo, Osaka, or Aichi Prefecture; 3) in daily practice; and, 4) under 60 years of age. This study was approved by the Ethics Committee of the Japanese Pediatric Society. Informed consent was obtained from all participants when they returned the completed questionnaires by mail.

The questionnaires were mailed to 532 pediatricians; the other 5 had moved. All subjects were asked to complete the questionnaire and return it by mail. In this study, sports drinks, ionic beverages, and oral rehydration solutions were collectively termed “ionic beverages” to minimize the redundancy of questions and to simplify analysis of the responses. Brand names are not mentioned below; we are sensitive to possible marketing issues. Instead, brief mentions of each brand are provided in the footnote to Table 1.

**Table 1:** The frequency of recommending ionic beverage, recommended brands, consultation of excessive consumption, and knowledge of health problems

	All participants *N* = 215	Pediatric specialists *N* = 182	Non-specialists *N* = 31	
Q1. The frequency of recommending ionic beverages				
Often Sometimes Infrequently Rarely Never	34 (15%) 91 (42%) 51 (24%) 28 (13%) 11 (5%)	27 (15%) 80 (44%) 44 (24%) 23 (13%) 8 (4%)	6 (19%) 10 (32%) 7 (23%) 5 (16%) 3 (10%)	NS
Q2. The brand names of ionic beverages which you recommend				
Brand A Brand B Brand C Brand D Brand E Other brands Anything Not particular	130 (48%) 47 (22%) 22 (10%) 16 (7%) 10 (5%) 4 (2%) 2 (1%) 46 (21%)	108 (59%) 43 (24%) 14 (8%) 12 (7%) 7 (4%) 4 (2%) 2 (1%) 40 (22%)	20 (65%) 2 (6%) 7 (23%) 3 (10%) 3 (10%) 0 0 6 (19%)	NS *P* < 0.05 *P* < 0.05 NS NS N/A N/A N/A
Q3. Consultation of excessive consumption of ionic beverages				
Yes No No answer	75 (35%) 139 (65%) 1 (0.5%)	68 (37%) 113 (62%) 1 (0.5%)	7 (23%) 24 (77%) 0	NS
Q4. Knowledge on the health problems due to excessive consumption of ionic beverages				
know in detail know know a little know very little do not know No answer	16 (7%) 96 (45%) 56 (26%) 35 (16%) 9 (4%) 1 (0.5%)	14 (8%) 84 (46%) 46 (25%) 29 (16%) 8 (4%) 1 (0.5%)	2 (6%) 12 (39%) 10 (32%) 6 (19%) 1 (3%) 0	NS

NS: not significant, N/A: not assessed

Brand A: An oral rehydration solution mainly sold in hospitals and pharmacies, containing energy 100 kcal/l, sodium ion 50 mEq/l, and potassium ion 20 mEq/l; Brand B: Several types are available. Some are categorized into soft drinks for infants, and others into oral rehydration solution, containing energy 160 kcal/l, sodium ion 35 mEq/l, and potassium ion 20 mEq/l; Brand C: One of the most popular sport drinks in Japan. Several types are available,, containing energy 250 kcal/l, sodium ion 21 mEq/l, and potassium ion 5 mEq/l;. Brand D: Oral rehydration powder prescribed in clinics and hospitals. Its solution can be available commercially, containing energy 130 kcal/l, sodium ion 60 mEq/l, and potassium ion 20 mEq/l;. Brand E: One of the most popular sport drinks in Japan, containing energy 190 kcal/l, sodium ion 17 mEq/l, and potassium ion 2 mEq/l;. Several types are available

The contents of the questionnaire were as below.

Q1. Do you recommend ionic beverages to infants?

often, sometimes, infrequently, rarely, never.

Q2. Please write the brand names of ionic beverages which you recommend to infants.

Q3. Have you ever been consulted from the parents for excessive consumption of ionic beverages of their infants?

no, yes.

Q4. Do you know about the health problems of infants due to excessive consumption of ionic beverages?

know in detail, know, know a little, know very little, do not know.

Q5. To those who answered “often” or “sometimes” to Q1.

Which situations do you recommend ionic beverages to infants?

vomiting and/or diarrhea, pyrexia, failure to thrive, after bath, hot weather,

after sweating, others.

Q6. To those who answered “often” or “sometimes” to Q1.

Do you warn caregivers for their children not to take ionic beverages excessively?

always, sometimes, infrequently, rarely, never.

We explored whether the responses differed between specialized pediatricians certified by the Japanese Pediatric Society and others. The chi-squared test was used to explore the significance of between-group differences; a *p*-value < 0.05 was considered to indicate statistical significance. We excluded missing or invalid data, and we did not analyze between-group differences if fewer than 10 responses were received from either group.

## Results

We received 215 valid responses (response rate, 40%) from 182 board-certified pediatric specialists and 31 non-specialists. Of all responders, 34 (15%) frequently recommended ionic beverages, and 91 (42%) did so sometimes (Table 1). Brand A was the most commonly recommended (Table 1). Responders who recommended such beverages frequently or sometimes indicated that patients usually presented with acute symptoms including vomiting, diarrhea, and pyrexia. Most respondents reported that they cautioned against excessive consumption (Table 2). Sixteen respondents (7%) reported that they were very familiar with the fact that health could be compromised by excessive consumption, and 96 (45%) replied that they were familiar to some extent with the potential problem (Table 1). Of the recommended beverages, the favorite brand for both specialists and non-specialists was Brand A. Brand B was recommended more frequently by specialists, while brand C was recommended more frequently by non-specialists (Table 1). We found no significant difference in the other variables between specialists and non-specialists (Table 1).

**Table 2:** Situation where the participants recommend ionic beverages and caution for excessive consumption

	Participants who recommend ionic beverages frequently or sometimes			
	All *N* = 125	Pediatric specialists *N* = 107	Non-specialists *N* = 16	
Q5. Situation where the participants recommend ionic beverages				
Vomiting and/or diarrhea Pyrexia After bath Hot weather After sweating Failure to thrive Others	122 (98%) 39 (31%) 1 (1%) 5 (4%) 6 (5%) 0 9 (7%)	104 (97%) 30 (28%) 1 (1%) 3 (3%) 5 (5%) 0 9 (8%)	16 (100%) 9 (56%) 0 2 (13%) 1 (6%) 0 0	NS P < 0.05 N/A N/A N/A N/A N/A
Q6. Caution for excessive consumption of ionic beverages				
Always Sometimes Infrequently Rarely Never	38 (30%) 26 (21%) 25 (20%) 22 (18%) 14 (11%)	31 (29%) 25 (23%) 22 (21%) 17 (16%) 12 (11%)	6 (38%) 1 (6%) 3 (19%) 5 (31%) 1 (6%)	NS

NS: not significant, N/A: not assessed

Of all respondents, 75 (35%) had been consulted on excessive consumption of ionic beverages (Table 1). Among them, non-specialists recommended beverages to patients with pyrexia more often than did specialists. We found no difference in terms of the frequency of cautioning against excessive consumption between specialists and non-specialists (Table 2).

## Discussion

We explored the attitudes of practicing members of the Japanese Pediatric Society toward consumption of ionic beverages by children. It became clear that most participants of this study have appropriate attitude towards ionic beverages among children. Approximately 60% of respondents stated that they recommended ionic beverages often or sometimes. The beverages most often recommended were those developed for oral rehydration (Brands A and B). Brands C and E, which are less suitable in this respect, were recommended infrequently (about 15% of the time). A 2008 survey of members of the Society of Ambulatory and General Pediatrics of Japan found that approximately 20% of respondents recommended inappropriate beverages such as Brands C and E [13]. In the present survey, the incidence of inappropriate recommendations was less.

Commonly, ionic beverages were recommended to treat acute symptoms including vomiting, diarrhea, and pyrexia. Cautions against excessive consumption were given by about half of the respondents who recommended ionic beverages either often or sometimes. Therefore, a simple recommendation that an ionic beverage should be consumed is unlikely to trigger excessive consumption. However, about 40% of respondents had been consulted by patients about excessive consumption, suggesting that such consumption is not unusual. About 60% of the participants were familiar with the health problems attributable to excessive consumption; the extent of awareness thus seems to be adequate. A large majority of respondents prescribed ionic beverages in a responsible manner.

We found that specialists recommended appropriate ionic beverages more often than did non-specialists. In contrast, non-specialists recommended ionic beverages to patients with pyrexia more often than did specialists, suggesting that specialists recommended more appropriate beverages than did non-specialists and were more sensitive to the possibility of over-consumption.

It remains unclear whether doctor recommendations influence the consumption of ionic beverages. A survey of sports drinks consumption in nursery school children found that doctors’ advice triggered such consumption in 65% of children, suggesting that the influence of doctors’ recommendations was strong [14]. In a study on how excessive soft drink consumption could be curbed, parents replied that they would reduce consumption if a doctor so recommended [15]. Although the cited work was performed in an effort to control caloric intake and the purpose thus differed from that of the present study, the cited work showed that doctors’ advice may alter the children’s consumption behaviors. Kawashita et al. found that most parents of 3-year-old children who consumed sports drinks stated that pediatricians had recommended the drinks; however, the consumption levels of these children were significantly lower than those of children whose parents had not received such recommendations [16]. Annual ionic beverage sales are increasing in Japan, and children are targeted in some promotions. The effects of ionic beverages on children, especially infants and young children, needs to be better understood. Appropriate advice by pediatricians is important in this context.

There are several limitations to our study. First, both the number of subjects approached and the questionnaire response rate were inadequate. It would be unwise to seek to apply our results to all populations of pediatricians. It is important to survey more pediatricians and increase the response rate. In particular, the proportion of non-specialist respondents in the present study was low. Thus, the power of our statistical analyses may be inadequate. We targeted members of the Japanese Pediatrics Society. However, presently, many doctors who treat children are not members of the Society. Those doctors must also be surveyed to more accurately evaluate children’s consumption of ionic beverages.

## Conclusions

The attitudes of pediatricians toward children’s consumption of ionic beverages were generally appropriate. The attitudes of pediatric specialists were more appropriate than those of non-specialists. To prevent excessive consumption, it is important that pediatric specialists impart correct information. Academic societies must encourage pediatricians to understand circumstances under which ionic beverages are required and the potential risks involved.

## Abbreviations

VB1D: Vitamin B1 deficiency
